# Using full agreement across multiple large language models for title-and-abstract screening in systematic reviews: a proof-of-concept

**DOI:** 10.1186/s13643-026-03228-4

**Published:** 2026-06-12

**Authors:** Frederic Hilkenmeier, Merle Stoltenberg, Christian Stierle

**Affiliations:** 1 Psychology School, Hochschule Fresenius, Hamburg, Germany; 2 Department of Health Psychology and Paedagogy, Rīga Stradiņš University, Rīga, Latvia

**Keywords:** Systematic reviews, Large language models, Abstract classification, Machine learning, Evidence synthesis

## Abstract

**Background:**

The exponential growth of scientific literature poses significant challenges for conducting systematic reviews, particularly in the labor-intensive title-and-abstract screening phase. This study examines the feasibility of using multiple large language models (LLMs) for title-and-abstract screening in systematic reviews.

**Methods:**

We propose and evaluate a full-agreement approach using three commercially available LLMs from different model families (ChatGPT, Gemini, and Claude), in which automated classification decisions are only accepted when all three models assign the same label. This approach was examined across six datasets to assess its effectiveness. A structured workflow was developed to support implementation without requiring specialized technical expertise. Additionally, a stop criterion was introduced to ensure that LLM-based classification is only applied when predefined performance thresholds are met.

**Results:**

Across the six datasets, approximately four in five abstracts received full agreement across models and could therefore be classified automatically. For this subset of abstracts, classification performance was consistently higher than that of previous automated approaches using LLMs, with statistically significant improvements in all prespecified performance metrics.

**Conclusions:**

A full agreement approach across three LLMs may offer a promising and conservative strategy for title-and-abstract screening in systematic reviews by automating concordant decisions while reserving human review for discordant cases. As a proof-of-concept, the present findings support this approach as a possible workflow for reducing screening burden in the face of continued growth in the scientific literature, although its broader generalizability remains to be established.

**Supplementary Information:**

The online version contains supplementary material available at 10.1186/s13643-026-03228-4.

## Introduction

The volume of published research articles has grown exponentially in recent years. In 2022, the number of published articles was 47% higher than in 2016 [30]. This trend is expected to continue, with annual growth rates projected between 4 and 8% [9, 10]. As scientific output increases, navigating and identifying relevant knowledge becomes increasingly difficult. Against this backdrop, evidence syntheses have become essential for summarizing research on specific topics (e.g., [20, 82]. Among these, systematic reviews are widely considered the “gold standard” of evidence synthesis. They play a crucial role in advancing scientific knowledge by consolidating evidence, identifying research gaps, and informing policy and practice (e.g., [2, 26, 27, 48]).

Unfortunately, conducting systematic reviews is inherently labor-intensive and time-consuming [2, 8, 38, 62, 81]. Estimates suggest that completing a review can take between 6 months and well over a year [8, 28] and cost in excess of $100,000 [46, 54]. However, the same rapid growth in the primary literature that increases the need for evidence syntheses also makes traditional systematic review methodologies increasingly difficult to sustain at scale [24, 38, 51]. As a result, important evidence gaps remain; for example, an analysis of lung cancer research found that only 10 to 17% of relevant treatment comparisons were covered by a comprehensive, up-to-date systematic review [19].

One critical bottleneck contributing to this problem lies in the initial screening of studies for relevance, which can take months to complete [46, 57, 62]. This stage serves “as a foundational step in curating the highest quality of evidence” ([48] p.3), the importance of which “cannot be overstated” ([73], p.1). Screening involves reviewing thousands or even tens of thousands of titles and abstracts to determine which studies meet predefined inclusion criteria. This process is not only cognitively demanding [69] even describe it as “overwhelming”), but also prone to inconsistencies and variability across human reviewers [8, 63, 81].

To reduce the risk of bias, improve consistency, and clarify ambiguous inclusion criteria, established guidelines—such as those from the Agency for Healthcare Research and Quality [52], Cochrane [31], the Joanna Briggs Institute [3, 64] or the Institute of Medicine [36]—therefore emphasize that title-and-abstract screening should be done by at least two raters, even though the associated additional costs are substantial ([36]; also see [23, 73]). Given the rapid growth of primary studies [9, 10, 30], these requirements—although methodologically sound—can pose significant barriers for smaller research groups, research groups from low- and middle-income countries, or research groups in underfunded research areas from conducting systematic reviews, simply due to constraints in manpower and resources ([6, 58] also see [8, 26] for related arguments).

In recent years, several technical tools such as Covidence, Rayyan, and Abstrackr have been introduced to ease this bottleneck. These tools aim to make the title-and-abstract screening process more manageable by incorporating features such as abstract organization, deduplication, key-term highlighting, or ranking studies based on likelihood of inclusion [40, 51, 60, 71, 79]. While these tools certainly streamline the review process, their primary utility is to facilitate the substantial manual work that remains necessary, not to replace the two-reviewer process, i.e., they assist rather than replace human reviewers and still necessitate significant human oversight to ensure that important studies are not overlooked [7, 25].

## LLMs in systematic reviews

The advent of large language models (LLMs)—such as OpenAI’s GPT-series models, Google’s Gemini, or Anthropic’s Claude—has introduced transformative potential for automating aspects of the systematic-review process (e.g., [2, 26, 50, 55, 78]). Trained on very large corpora of unstructured text, LLMs can process natural-language instructions and generate text-based outputs across a wide range of tasks. Rather than following fixed, task-specific rules, they generate outputs on the basis of learned statistical patterns in language, which makes them highly flexible but also inherently probabilistic rather than fully deterministic systems [21, 41, 72]. Unlike more traditional machine-learning approaches, which typically require structured training data and model development for a specific application, LLMs can thus be adapted to new tasks through natural-language prompting rather than retraining [18, 21, 72].

This flexibility, together with the comparatively low technical barrier to deployment, differentiates LLMs from many earlier automation approaches in evidence synthesis. Whereas previous automation tools were often available only as research prototypes and difficult to sustain in routine use [51], LLMs can now be integrated into workflows through commercially provided platforms that were not necessarily developed for evidence synthesis but can nevertheless be adapted to such tasks through prompting [18, 72]. Recent work has explored their use for tasks such as search-term generation and refinement (with limited success, see [79]), full-text screening (with mixed results, see [41]), full-text information extraction (effective only as an assistive tool, see [26, 32], risk-of-bias analysis (promising, but not sufficient for systematic reviews, see [74] and drafting narrative summaries (with little success, see [4]).

As these studies indicate, LLMs are not a “silver bullet” for automation in evidence synthesis, a sentiment shared by [16] in their recent systematic review on LLM-usage in systematic reviews. Many of the observed shortcomings are consistent with the fundamental architecture of general-purpose LLMs: because they generate plausible outputs from learned statistical patterns rather than applying explicit methodological rules, they may hallucinate, fabricate, or over-infer information, omit relevant studies or details, and misrepresent or oversimplify source content ([18, 67, 74] also see [15]). Such problems have been reported, for example, when LLMs missed substantial proportions of relevant studies during searching or generated unsupported details during data extraction, including fabricated numerical values or inferred study characteristics not reported in the source text [13, 18, 39, 70, 83]. These task-level errors are further complicated by the limited transparency of many proprietary systems, including incomplete information about their training data, which makes it difficult to trace how specific outputs were generated and to assess them in a fully auditable and reproducible way [21, 66]. Consequently, current guidance increasingly frames LLMs as tools that require human oversight, transparent reporting, and responsible integration into evidence-synthesis workflows [55, 75].

### LLMs in title-and-abstract screening

Among the different stages of the systematic-review process, title-and-abstract screening appears to be a particularly plausible use case for LLMs—and the most frequently explored to date [16, 69]. Compared with tasks such as full-text synthesis or open-ended evidence generation, title-and-abstract screening typically involves relatively short and standardized inputs and a comparatively straightforward include/exclude decision based on prespecified criteria. In this sense, the task is less affected by performance degradation associated with longer or more complex input contexts [49] and less reliant on the kind of open-ended generation in which hallucinated content is more likely to arise [16, 35, 37]. Moreover, titles and abstracts can be evaluated in a zero-shot manner, that is, without task-specific (re-)training of the underlying model [80]. Because each abstract is processed independently under the same prompt, prior classification decisions do not influence subsequent ones, thereby removing one source of inconsistency that can arise in human screening when judgments shift over time as reviewers implicitly recalibrate inclusion and exclusion criteria [5, 44]. From a workflow perspective, this creates the potential to categorize thousands or even tens of thousands of titles and abstracts within hours, a process that would take human reviewers several months [46, 57].

This theoretical plausibility is only partly reflected in the empirical literature. Studies using a single LLM for title-and-abstract screening have reported highly variable results across datasets and review topics (see Table S1 in the Electronic Supplementary material for an overview and reanalysis based on Scherbakov et al.’s [69] systematic review). A recurring pattern across these studies is that single-LLM approaches often achieve relatively high recall (sensitivity), sometimes even approaching perfect recall, but at the cost of low precision and specificity. In the context of systematic reviews, recall is often prioritized to minimize the risk of missing relevant studies [22]. However, the results summarized in Supplementary Table S1 suggest that single LLMs also identify large numbers of irrelevant records as potentially relevant, thereby substantially limiting their practical utility for streamlining review workflows [63]. Across the reanalyzed studies, the unweighted mean precision was only 0.31, indicating that fewer than one in three records classified as relevant by a single LLM were in fact relevant. This low precision reduces efficiency at later review stages, since researchers must still spend considerable time screening irrelevant full-text articles and extracting information from records that should ideally have been excluded earlier [26, 63].

Further limitations of single-LLM approaches can be seen in the Krippendorff’s alpha scores, which measure agreement with human consensus-based screening decisions while accounting for chance. As shown in Supplementary Table S1, Twenty-nine of the 30 alpha-scores are below 0.67—and should therefore not be relied upon, and none exceeds the commonly accepted threshold of 0.8 for strong reliability ([45, 53] see Krippendorf, [44], for a more detailed discussion). Such levels of reliability would be unacceptable among human reviewers in a standard title-and-abstract screening process, and they are equally unacceptable for an LLM intended to replace a human reviewer. Taken together, these results suggest that single LLMs are currently of limited use in title-and-abstract screening. While they may accelerate parts of the workflow, this comes at the cost of limited precision, weak agreement with human screening decisions, and a high number of false-positive classifications, which in turn slow down subsequent stages such as full-text screening and information extraction.

A logical next step is to combine multiple LLMs rather than relying on a single model. Li et al. [48], and more recently [33], explored such approaches by processing titles and abstracts with multiple readily available LLMs (e.g. OpenAI’s ChatGPT, Meta’s Llama, Microsoft’s Phi) and aggregating their independent classification decisions. Although the concrete implementations were more complex than described here, both studies included a setup in which multiple LLMs evaluated the same abstract independently. Interestingly, despite receiving identical inputs, agreement between the different LLMs varied considerably, with Krippendorff’s alpha scores ranging from 0.42 to 0.73. Had such levels of agreement been observed between human reviewers, they would likely have prompted additional calibration or training. Despite this comparatively low inter-LLM agreement, final classifications were still derived by aggregating the LLMs’ decisions. As shown in Table 1, this aggregation approach improves performance over using a single LLM. While recall (sensitivity) remains high, precision and specificity increase, suggesting that combining multiple LLMs reduces the tendency of a single model to misclassify irrelevant articles as relevant. However, the empirical basis remains limited, as these findings are based on three reanalyzed datasets from [48] and one reanalyzed dataset from [33], supplemented by two additional datasets introduced by the present study and presented in more detail below. Despite these improvements, Krippendorff’s alpha scores suggest that reliability remains a concern. Of the six datasets (re-)analyzed, only two achieved a strong level of agreement between the LLM majority vote and human consensus-based screening decisions. Given the crucial role of systematic reviews in consolidating evidence, identifying research gaps, and informing policy decisions (e.g., [2, 26, 27, 48]), these results remain insufficient to meet the rigorous demands of systematic reviews.

**Table 1:** Performance of multi-LLM aggregation approaches for title-and-abstract screening

Study	LLM-type	model specification (detailed in original article)	Dataset (detailed in original article)	Precision	Recall/Sensitivity	Specificity	F1	Accuracy	Krippendorff’s alpha
				Proportion of true positive results among all positive predictions	Proportion of true positive cases correctly identified among all actual positive cases	Proportion of true negative cases correctly identified among all actual negative cases	Harmonic mean of precision and recall, balancing their trade-off in a single metric	Proportion of all correct predictions among the total number of cases	Reliability measure assessing the agreement among raters (“human consensus-based screening decisions” vs. “LLM majority vote classifications”), accounting for chance agreement
Reanalysis of Li et al., 2024 [48]	GPT 3.5; GPT 4.0; Llama	Majority Voting	Bannach- Brown 2016	0,88	0,96	0,87	0,92	0,92	0,83
Reanalysis of Li et al., 2024 [48]	GPT 3.5; GPT 4.0; Llama	Majority Voting	Meijboom 2021	0,46	1,00	0,63	0,63	0,72	0,41
Reanalysis of Li et al., 2024 [48]	GPT 3.5; GPT 4.0; Llama	Majority Voting	Menon 2022	0,91	0,81	0,94	0,86	0,84	0,76
Reanalysis of Hu et al., 2025 [33]	GTP 4o; Llama 3.1; Phi 4	Ensemble Agent (Majority Voting)	GREP‐Agent Workflow after fine-tuning (measles)	0,89	0,84	0,94	0,86	0,90	0,78
current article	GPT 4.0; Gemini Pro 1.0; Claude 3 Sonnet	Majority Voting	471 titles and abstracts with human consensus-based screening decisions	0,78	0,92	0,96	0,85	0,95	0,82
current article	GPT 4.0; Gemini Pro 1.0; Claude 3 Sonnet	Majority Voting	2499 titles and abstracts with at least one human rating	0,58	0,93	0,92	0,72	0,92	0,67

### Using full agreement across multiple LLMs for title-and-abstract screening

To meet these rigorous demands and address the low inter-LLM reliability, we propose a more conservative approach: employing multiple independent LLMs but only accepting classification decisions when all models assign the same label, that is, when they are in full agreement. Naturally, restricting automated decisions to cases of full agreement reduces the amount of work saved over sampling (e.g., [17, 60]) compared to a majority-vote approach. This lower efficiency must therefore be offset by superior performance across key metrics to ensure that the full agreement approach provides a clear advantage.

**Fig. 1:**
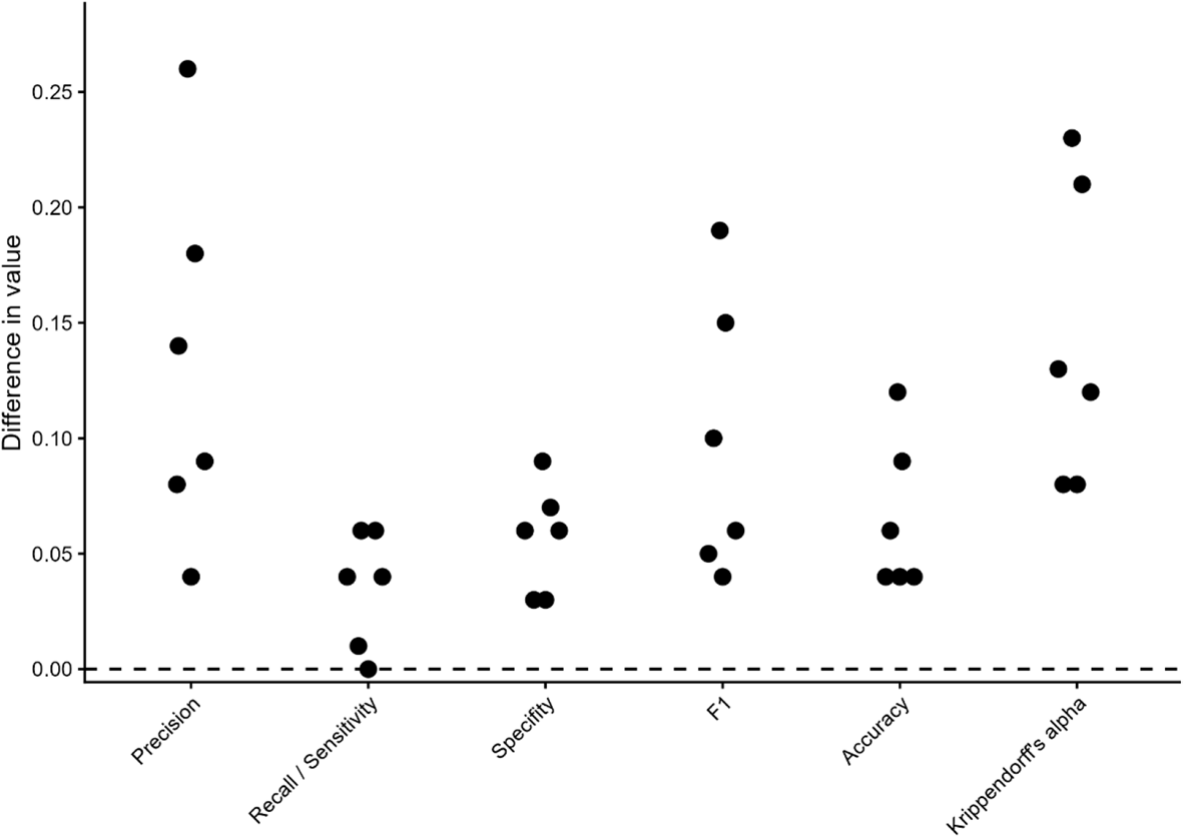
Analysis of key metric value differences between the full agreement approach and the majority-vote approach across the six datasets detailed in Tables 1 and 2. Positive differences indicate superior performance of the full agreement approach

Fortunately, the raw data used in the analyses by [48] and [33] are publicly available. This allowed us to apply our full agreement approach not only to our two new datasets (detailed below), but also to the four datasets used by [48] and [33]. Therefore, we were able to compare the majority-vote approach with the full agreement approach across six datasets in total. As demonstrated in Table 2 and Fig. 1, this trade-off leads to better performance across all key metrics. Statistical validation further supports these findings. One-tailed paired t-tests comparing the metrics in Tables 1 and 2 yield *p*-values < 0.05, confirming a statistically significant improvement. Even the Wilcoxon test and the more conservative sign test show significant one-tailed results for all relevant metrics, despite the limited sample size (*n* = 6).

**Table 2:** Performance of the proposed full agreement approach across three different LLMs for title-and-abstract screening

Study	LLM-type	Dataset (detailed in original article)	Proportion with full agreement	Precision	Recall/Sensitivity	Specificity	F1	Accuracy	Krippendorff’s alpha
			Proportion of titles and abstracts with full agreement. All other values are calculated using this subset	Proportion of true positive results among all positive predictions	Proportion of true positive cases correctly identified among all actual positive cases	Proportion of true negative cases correctly identified among all actual negative cases	Harmonic mean of precision and recall, balancing their trade-off in a single metric	Proportion of all correct predictions among the total number of cases	Reliability measure assessing the agreement among raters (“human consensus-based screening decisions” vs. “LLM full agreement classifications”), accounting for chance agreement
Reanalysis of Li et al., 2024 [48]	GPT 3.5; GPT 4.0; Llama	Bannach- Brown 2016	0.79 (157 of 200 titles and abstracts)	0,96	0,97	0,94	0,96	0,96	0,91
Reanalysis of Li et al., 2024 [48]	GPT 3.5; GPT 4.0; Llama	Meijboom 2021	0.57 (75 of 132 titles and abstracts)	0,64	1,00	0,72	0,78	0,81	0,62
Reanalysis of Li et al., 2024 [48]	GPT 3.5; GPT 4.0; Llama	Menon 2022	0.61 (106 of 173 titles and abstracts)	1,00	0,85	1,00	0,92	0,96	0,89
Reanalysis of Hu et al., 2025 [33]	GTP 4o; Llama 3.1; Phi 4	GREP‐Agent Workflow after fine-tuning (measles)	0.82 (1633 of 1998 titles and abstracts)	0,93	0,88	0,97	0,91	0,94	0,86
current article	GPT 4.0; Gemini Pro 1.0; Claude 3 Sonnet	402 abstracts with full agreement and human consensus-based screening decisions	0.85 (402 of 471 titles and abstracts)	0,92	0,98	0,99	0,95	0,99	0,94
current article	GPT 4.0; Gemini Pro 1.0; Claude 3 Sonnet	2049 abstracts with full agreement and (at least) one human rating	0.82 (2049 of 2499 titles and abstracts)	0,84	0,99	0,98	0,91	0,98	0,90

## A proposed workflow

Taken together, these findings support limiting automated title-and-abstract screening decisions to cases of full agreement across three independent general-purpose LLMs. Under this approach, human reviewers are involved only in a subset of titles and abstracts at two critical points:

- Multiple human reviewers independently screen approximately 10–20% of titles and abstracts and resolve disagreements to establish consensus-based screening decisions that serve as a calibration and evaluation benchmark for the LLM workflow.
- At least one human reviewer adjudicates titles and abstracts for which the LLMs do not reach full agreement, thereby making the final screening decision in cases not classified automatically.

**Fig. 2:**
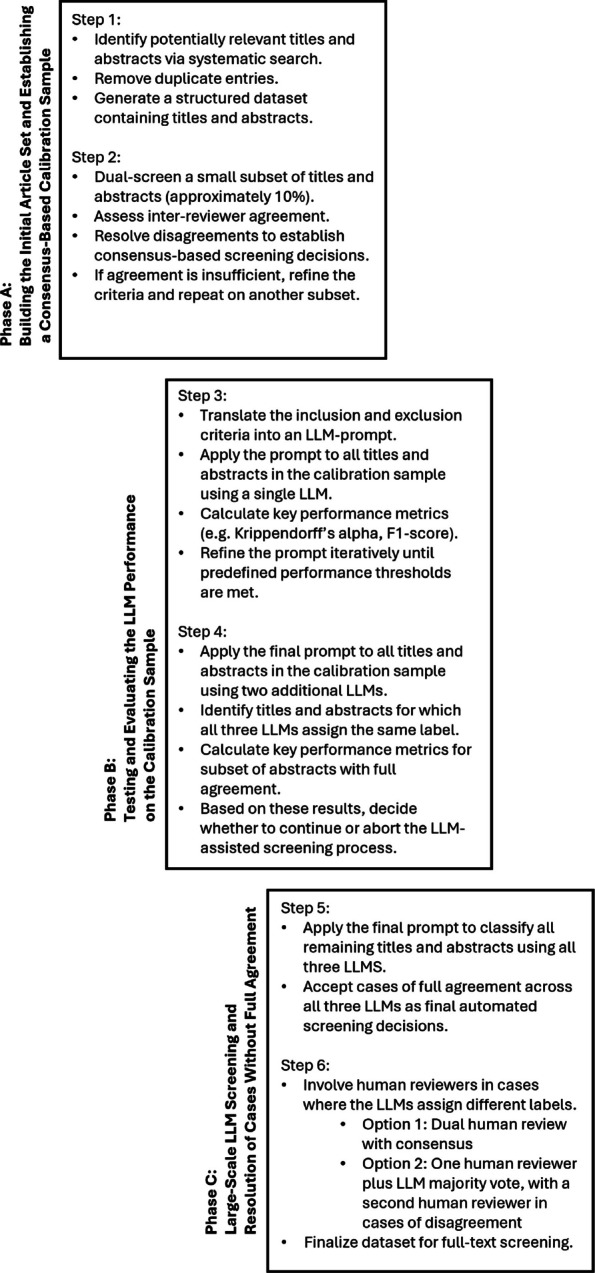
Proposed workflow for conservative LLM-assisted title-and-abstract screening

In the following, we describe the proposed workflow in more detail, using a continuous example from our own systematic review case study (in preparation) to illustrate each step. This example serves as a guiding thread throughout this section, making the workflow easier to follow and potentially adaptable for other review teams seeking to evaluate or further validate this approach. The procedure is intended as an accessible implementation model, requiring no specialized technical expertise or task-specific training data. A key feature of this approach is its built-in “stop criterion”, which allows review teams to assess early in the process whether the method is performing adequately for their specific review. If performance is insufficient, they can revert to manual title-and-abstract screening, with only limited loss of time and resources. As shown in Fig. 2, human oversight, verification, and accountability are embedded at multiple stages of the screening process, thereby providing a conservative implementation model for LLM-assisted title-and-abstract screening that reflects the current emphasis on responsible, transparent, and accountable AI use in evidence synthesis [2, 34, 42, 55, 69, 75, 78].

### Phase A: building the initial article set and establishing a consensus-based calibration sample

#### Step 1: identifying potentially relevant articles

The search strategy remains unchanged under this workflow. Researchers conduct systematic searches across relevant databases using prespecified keywords and queries (e.g., [47, 68, 79]) and remove duplicate citations (e.g., [65]). In line with PRISMA 2020 terminology, we refer to the titles and abstracts screened at this stage as records [61]. At the end of Step 1, researchers should have a structured dataset (e.g., a.csv file) containing the titles and abstracts of all potentially relevant records.

In our case, we obtained a.csv file containing the titles and abstracts of 2514 unique records.

#### Step 2: dual-screening a calibration subset, refining the criteria, and reaching consensus

This “calibration process” [52] also remains unchanged under this workflow. For instance, [52] recommend pairing a methodologist with a subject-matter expert whenever possible. The Institute of Medicine [36] cites Buscemi et al. [12], emphasizing that including a third experienced reviewer can help reach consensus and reduce false classifications. Cochrane guidelines [31] suggest applying the PICO framework (Population, Intervention, Comparison, Outcome) as guardrails for inclusion and exclusion criteria to minimize risk of bias and human error in the title-and-abstract screening process. Before reaching consensus among reviewers, it is essential to assess interrater reliability to ensure that all reviewers apply the inclusion and exclusion criteria consistently. As Belur et al. ([5] p. 841) note, reviewers must “share the [same] mental schema in order to achieve both consistency and accuracy of coding.” Low interrater reliability may indicate unclear or inconsistent criteria and should be addressed before proceeding. Agreement coefficients above 0.8 are generally considered acceptable [29, 44, 45, 53]. If agreement is below this threshold, reviewers should undergo additional training, and reliability should be reassessed. At the end of Step 2, researchers should have generated a dataset (e.g., a.csv file) containing the title, abstract, and consensus-based screening decision for each reviewed record.

In our case example, Two hundred forty-nine of all 2514 records were independently reviewed by three reviewers: an expert in the field, a methodologist without subject-matter expertise, and a research assistant. For these 249 records, Krippendorff’s alpha was 0.48, indicating unacceptably low agreement. As a result, the reviewer conferences scheduled to reach consensus were also used to further refine and calibrate the inclusion and exclusion criteria used in this study.

#### Step 2b: dual-screening an additional subset of titles and abstracts and reaching consensus

Step 2b is only required if the interrater reliability from Step 2 falls below the desired threshold of 0.8. This step ensures that reviewers apply the inclusion and exclusion criteria consistently and with a shared understanding. The number of additional records to review depends on the total dataset size. However, an incremental approach—reviewing records in smaller batches and repeatedly evaluating interrater reliability—is recommended until the desired agreement level is reached. At the end of Step 2b, researchers should have generated another dataset (e.g., a. csv file) which contains title, abstract, and consensus-based screening decision for each unique record reviewed in Step 2b.

In our case example, after further training, the three reviewers independently reviewed another 222 records. For these 222 records, Krippendorff’s alpha reached 0.82, indicating strong agreement (e.g., [29]). Afterwards, a consensus decision was reached for all 222 records. The classification data from Step 2 and Step 2b were then merged into a single.csv file, containing the title, abstract, and consensus-based screening decision for 471 records. (Again, had the interrater reliability from Step 2 been sufficient, Step 2b would have been skipped.)

### Phase B: testing and evaluating the LLM performance on the calibration sample

#### Step 3: translating the inclusion/exclusion criteria into a prompt, applying a single LLM to the calibration sample, calculating key performance metrics, and repeating the process

Using the refined inclusion and exclusion criteria from Step 2 or Step 2b, the next step is to formulate a clear prompt instructing the LLM (via API) to classify each record in the calibration sample as either “potentially relevant” or “irrelevant”. This process is conducted in a zero-shot manner, meaning each record is evaluated in isolation without guiding examples [80]. Various prompting strategies exist (e.g., [14, 43]), with Chain-of-Thought prompting being a particularly promising approach [77]. At the same time, prompt development should not be understood as a one-time setup. As Colangelo et al. ([18] p. 11) note, “Prompt engineering is seldom perfected in one pass, so an iterative process of testing, refining, and retesting the prompts with a small validation set is often very important to identify where false positives and false negatives occur and to recalibrate the instructions accordingly”. In line with this recommendation, we apply the prompt to all records in the calibration sample, compute Krippendorff’s alpha and the F1-score as key indicators of agreement and classification performance, and then iteratively refine the prompt until both metrics exceed 0.8. Of course, researchers may choose to prioritize different metrics or thresholds depending on the specific needs of their review. At the end of Step 3, researchers have developed a calibrated prompt that shows strong agreement with the human consensus decisions in the calibration sample.

Since the core of our proposed workflow begins at this step, our case example will from now on be described in more detail. While our approach may not be the most sophisticated, it is intentionally designed to remain accessible and to require only limited technical expertise. In our case example, this step and the subsequent steps were implemented using the following resources:

- A free Google account to access Google Sheets.
- API keys for OpenAI’s ChatGPT, Anthropic’s Claude, and Google’s Gemini, which can be generated after registration.
- The Chrome browser and the Chrome extensions “GPT for Sheets and Docs”, “Claude for Sheets”, and “AI Assist for Gemini in Sheets and Docs”.
- A valid credit card (required for LLM API usage).

Researchers with greater technical expertise could streamline the same process in Python or R, but such programming expertise is not required. To ensure replicability, we outline one possible step-by-step implementation using Google Sheets. We start with the.csv file from Step 2b, which contains 471 rows, each representing a unique record. The file is structured as follows: Column A contains the prompt. Column B contains the respective title. Column C contains the respective abstract text. Column D contains the respective human consensus-based screening decision.

We uploaded this.csv file to Google Sheets and used the “GPT for Sheets and Docs” plugin, which integrates OpenAI’s GPT models directly into the spreadsheet, enabling automated classification. Using this setup, GPT 4.0 processed each record individually, retrieving information from Columns A, B, and C for each row (1 to 471) and storing the classification decision in Column E. In our case example, this initial classification step required only a few minutes.

Within the spreadsheet, we then calculated true positives (TP), true negatives (TN), false positives (FP), and false negatives (FN) based on the human consensus-based screening decisions (Column D) and the LLM classification (Column E). Finally, we computed Krippendorff’s alpha and the F1-score using the following formulas:

$$\upalpha =1-\frac{(FP+FN)(TP+TN+FP+FN)}{(TP+FP)(FP+TN)+(TP+FN)(FN+TN)}$$

$$F1=2\times \frac{TP}{2\times TP+FP+FN}$$

In our case example, achieving an F1-score of 0.85 and a Krippendorff’s alpha of 0.83 took 22 iterations on the initial prompt, which we consider rather extreme. However, up to that point, we had not yet engaged with the literature on prompt engineering and approached the task somewhat naively. The entire process, nevertheless, was completed in several hours and incurred API costs in the low two-digit dollar range.

#### Step 4: applying the final prompt to two additional LLMs on the calibration sample and calculating key performance metrics

This optimized final prompt is now applied to classify all records in the calibration sample using two additional LLMs (see [48]). Ideally, these LLMs should come from different model families, as LLMs from distinct architectures often produce different classification decisions, even when provided with the same input (see [48, 59]).

In this step, the two additional LLMs receive only the final prompt, along with the title-and-abstract text, but not the consensus-based screening decision or the classification decision of the first LLM. After classification, each record in the calibration sample now has three independent classification decisions from three different LLMs. Next, we calculate the proportion of records for which all three LLMs assign the same label (full agreement rate), as well as key performance metrics for this subset of records with full agreement.

These performance results also serve as a “stop criterion”: If the key performance metrics for records with full agreement are too low (e.g., Krippendorff’s alpha < 0.8, F1-score < 0.9), the LLMs do not classify the calibration sample with sufficient consistency, making the approach unsuitable for the given review context.

This built-in stop criterion is crucial: it allows review teams to assess early on whether this approach is performing adequately for their dataset. If the results are unsatisfactory, the review team can discontinue the process, having only invested a few hours of work and a relatively modest amount of API cost.

In our case example, we continued working with the Google Sheets file from Step 3, which contains 471 rows. To expand the classification process, we installed the additional commercial Chrome plugins “Claude for Sheets” and “AI Assist for Gemini in Sheets and Docs”.

Next, we instructed Claude 3 Sonnet to process each row individually, retrieving information from Columns A, B, and C and writing its classification decision in Column F. Similarly, Gemini Pro 1.0 processed the same data and recorded its classification in Column G. In our case example, this procedure took less than an hour.

Within Google Sheets, we then calculated the proportion of records for which all three LLMs—GPT 4.0, Claude 3 Sonnet, and Gemini Pro 1.0—reached an identical classification decision (full agreement).

In our case example, 402 records, i.e., about 85% of all 471 records were classified with full agreement. For these 402 records, we calculated Krippendorff’s alpha and the F1-score using the same formulas as before. The results indicate almost perfect agreement with the consensus-based screening decisions, Krippendorff’s alpha = 0.94 and F1-score = 0.95. As shown in Table 3, the full agreement classification deviated from the consensus-based screening decisions in only 5 of 402 cases. Notably, only one relevant abstract was falsely excluded by the LLMs. The resulting key performance metrics, such as precision and accuracy, are reported in the fifth row of Table 2.

**Table 3:** Cross tabulation of consensus-based screening decisions and full agreement classification for the calibration dataset with 402 records

	Consensus-based screening decision: include	Consensus-based screening decision: exclude
Full agreement classification: include	49	4
Full agreement classification: exclude	1	348

Again, had the results been different, with e.g., Krippendorff’s alpha < 0.8, we would have concluded that the full agreement classifications did not align sufficiently with the consensus-based screening decisions and would not have proceeded with LLM-assisted title-and-abstract screening for this particular review.

### Phase C: large-scale LLM screening and resolution of cases without full agreement

#### Step 5: applying the final prompt to all three LLMs for all titles and abstracts not included in the calibration sample

So far, LLM classification has only been applied to the 10 to 20% of records with consensus-based screening decisions. If Step 4 confirms a strong agreement between the consensus-based screening decisions and the full agreement classifications, the next step is to extend the classification process to all remaining records.

The three LLMs independently classify the remaining records using the final prompt from Step 3 in a zero-shot manner. Depending on the number of records, this step may require several hours of computing time and substantial token costs. For each record with full agreement, this decision is accepted as the final automated screening decision.

In our case example, we return to the.csv file from Step 1, which contains the titles and abstracts of all 2514 unique articles. We removed the 471 records that were part of the calibration sample and already had consensus-based screening decisions and LLM classifications, leaving 2043 records.

The updated file was uploaded to Google Sheets, and the final prompt from Step 3 was inserted in Column A. The three LLMs—GPT 4.0, Claude 3 Sonnet, and Gemini Pro 1.0—were instructed to classify each record using the same method as before. The process required several hours and incurred a cost of approximately $100 in token usage.

In our case, 1647 out of 2043 records (≈ 81%) received full agreement, closely aligning with results from Step 4. Notably, Gemini Pro 1.0 refused to classify 15 records due to the presence of sensitive terms such as “incest,” “pedophilia,” and “child trafficking”. This suggests that content filtering prevented the model from processing such requests. As a result, we obtained full LLM classification data for 2499 records.

#### Step 6: resolving the remaining cases without full agreement

So far, consensus-based screening decisions have been established for approximately 10–20% of all records (Steps 2 and 2b), and full agreement classifications have been obtained for the majority of records not included in the calibration sample (Step 5). However, the remaining cases without full agreement still require a final classification decision.

In our view, this can be resolved using one of two approaches. The default option follows the traditional dual human review, in which two human reviewers independently classify the remaining records and resolve disagreements through consensus, as done in Steps 2 and 2b. The second option is an optional hybrid approach, in which a single human reviewer classifies the remaining records while the LLM majority-vote [48] serves as the second reviewer. If the human reviewer disagrees with the LLM majority-classification, a second human reviewer is consulted to reach a final consensus. While this hybrid approach may be considered controversial, it may offer a resource-efficient alternative. Given the comparatively strong performance metrics of the LLM majority-vote, as shown in Table 1, this method may maintain a high classification quality, particularly since the human review remains decisive in cases of disagreement. Previous research suggests that this approach can be viable under specific conditions [1, 56, 69, 76].

In our case example, we started with 2514 records in total. Of these, 471 records were classified via human consensus in Steps 2 and 2b, while 1647 records received full agreement classification in Step 5. This left 396 records that remained without full agreement and required further classification. To resolve these cases, the methodologist (Reviewer 2) created a new file containing only the 396 remaining records, including the title and abstract text but excluding LLM classifications. The research assistant (Reviewer 3) then independently classified these records. Next, Reviewer 2 compared Reviewer 3’s classifications with the LLM majority-vote. In 120 cases, the classifications did not align. For these cases, the expert in the field (Reviewer 1) provided input on the final classification decision. Using this workflow, the total manual workload was reduced by over 70%: in a traditional approach, each of the 2514 records would have required classification by two human reviewers, resulting in 5028 manual classifications. In contrast, the proposed workflow required only 1458 manual classifications (471 records classified by two reviewers, 396 classified by one reviewer, and 120 requiring an additional expert review). This demonstrates that integrating LLMs into the screening process can significantly reduce the workload while maintaining high classification reliability.

### Supplementary validation analysis

As a supplementary analysis, Reviewer 3 also classified the remaining 1647 records that had received full agreement classifications, but no human screening decision. It is important to note that this classification does not establish a new consensus-based benchmark, as it reflects the decision of a single reviewer rather than a consensus-based classification. However, for the purpose of this additional analysis only, we treated this classification as a single-reviewer reference decision and merged these 1647 records with the 402 records from Step 4, which already had both human consensus-based screening decisions and full agreement classifications.

As shown in Table 4, the full agreement classifications deviated from the single-reviewer reference decisions in only 46 of 2049 cases. Notably, only two relevant records were falsely excluded by the LLMs. The resulting key performance metrics, such as precision and accuracy, are reported in the sixth row of Table 2. Taken together, these results provide additional support for the proposed method, as they demonstrate a strong alignment between the full agreement classifications and a single human reviewer’s decisions.

**Table 4:** Cross tabulation of single-reviewer reference decisions and full agreement classifications for dataset with 2049 records

	Single-reviewer reference decision: include	Single-reviewer reference decision: exclude
Full agreement classification: include	235	44
Full agreement classification: exclude	2	1768

However, review teams should consider this final validation step mainly for further evaluation of the method, rather than for its routine use, because it substantially reduces the resource savings achieved through the LLM-assisted screening process.

## Discussion and conclusion

Across the six datasets examined, limiting automated title-and-abstract screening decisions to cases of full agreement across three independent LLMs was associated with consistently better performance than previously used automated approaches. At the same time, these gains came at the cost of a reduced proportion of automatically classified records. The central question raised by this study is whether the loss in automated coverage is offset by a sufficient gain in classification quality. The present results suggest that this is the case (see Table 2; Fig. 1).

This pattern is particularly relevant in light of the limitations of earlier LLM-based approaches to title-and-abstract screening. Previous studies using a single LLM often reported high recall but low precision and specificity, leading to substantial overclassification of irrelevant articles and thereby reducing efficiency at later review stages (e.g., [69, 76] Supplementary Table S1). Majority-vote approaches improved on this pattern, but performance remained inconsistent across datasets and, in several cases, still fell short of the level of agreement with human screening decisions required for systematic reviews ([33, 48] Table 1). Against this background, the present findings suggest that full agreement may provide a more conservative way of using LLMs for screening. Automated decisions are accepted only when all three LLMs assign the same label, whereas discordant cases remain subject to human review. This approach adapts the consensus principle used in traditional systematic reviews to an LLM-assisted screening context: greater confidence in a screening decision should be associated with agreement across independent assessments [36, 52]. By prioritizing robustness over maximal automation, the approach may be particularly relevant in review contexts where false-positive and false-negative screening decisions have substantial downstream consequences for full-text screening, evidence extraction, and synthesis.

The workflow proposed in this study may offer a practical way of implementing this conservative decision rule while preserving human oversight at critical stages. Rather than treating LLM-assisted screening as appropriate by default, the workflow requires early evaluation on the calibration sample and restricts broader automation to review contexts in which predefined performance thresholds are met on that sample. Its built-in stop criterion therefore functions as a methodological safeguard: if performance on the calibration sample is insufficient, review teams can discontinue the LLM-assisted procedure and revert to conventional human screening. This safeguard can be illustrated retrospectively using the Meijboom 2021 dataset reanalyzed from [48]. In that dataset, the performance of the full agreement approach remained below the predefined thresholds, with both Krippendorff’s alpha and the F1-score failing to reach the required levels. Had the proposed workflow been applied in that review context, the process would therefore likely have been discontinued after Step 4, and the remaining records would have proceeded to conventional human screening rather than LLM-assisted screening. This retrospective example illustrates the intended function of the stop criterion: to prevent broader use of the workflow in datasets for which the required level of reliability is not achieved.

Several limitations should nevertheless be acknowledged. First, the performance of the proposed workflow depends substantially on prompt design and prompt calibration. In our case example, 22 prompt iterations were required before the predefined performance thresholds were met. Although this process was feasible within a relatively short time frame, it also indicates that the workflow is sensitive to how inclusion and exclusion criteria are operationalized in the prompt. Future research should therefore examine whether alternative prompting strategies, including few-shot prompting—that is, providing the model with a small number of labeled examples within the prompt [11, 18]—or improved prompt calibration can reduce this setup effort and improve robustness across review contexts (see [14, 43, 77]).

Second, the efficiency gains of the proposed workflow are likely to vary across review contexts. Topics with more ambiguous eligibility criteria, more heterogeneous abstracts, or more borderline cases may yield lower full-agreement rates and therefore smaller reductions in manual screening effort. The approach is therefore best understood not as a replacement for reviewer judgment, but as a conservative model for selectively automating those screening decisions for which full agreement across models is achieved.

Third, the broader generalizability of the present findings remains limited. The study examined six datasets, including reanalyzed datasets from prior work and two additional datasets from our own case study, but this evidence base remains relatively small. Further evaluation across additional review topics, disciplines, implementation settings, and non-English records is therefore needed [41]. In addition, because the workflow relies on proprietary general-purpose LLMs, future work should examine how version changes, content-moderation behavior, and other platform-specific constraints may affect reproducibility across review settings and over time.

At the same time, if satisfactory performance on the calibration sample can be established, the proposed workflow may be particularly relevant for review teams working under limited time or personnel resources, because it concentrates human effort on calibration and discordant cases rather than requiring dual human screening of all records ([6, 8, 58] see also [62]). This emphasis on continued evaluation is also reflected in recent responsible-AI guidance for evidence synthesis, which highlights the importance of validation studies to inform best-practice guidance and ensure that emerging tools are fit for purpose (e.g., [75]). Given the rapid pace of development in generative AI, such evaluation remains essential.

In conclusion, this study suggests that limiting automated title-and-abstract screening decisions to cases of full agreement across three independent LLMs may offer a promising and conservative approach to LLM-assisted screening in systematic reviews. Across the six datasets examined, the approach was associated with better performance than single-LLM screening and majority-vote aggregation, albeit for a reduced subset of automatically classified records. The proposed workflow is therefore best understood not as a replacement for human reviewers, but as a proof-of-concept for reducing screening burden while preserving human oversight at critical stages.

## Supplementary Information

Supplementary Material 1. Supplementary Table S1: Overview of studies using a single LLM for title-and-abstract classification.

## Data Availability

The datasets used and/or analyzed during the current study are available from the corresponding author on reasonable request.
