# Supplementary material for: Using full agreement across multiple large language models for title-and-abstract screening in systematic reviews: a proof-of-concept
Source: Syst Rev. 2026 Jun 12;15:191. doi: 10.1186/s13643-026-03228-4 (PMC13263951; doi:10.1186/s13643-026-03228-4)
Supplement: Supplementary file 1 — Supplementary Material 1. Supplementary Table S1: Overview of studies using a single LLM for title-and-abstract classification. [file 13643_2026_3228_MOESM1_ESM.docx]

| **Overview of studies using a single LLM for title-and-abstract classification**  **Reanalyses based on Scherbakov et al.’s (2024) systematic review** | | | | | | |  |  |  |
| --- | --- | --- | --- | --- | --- | --- | --- | --- | --- |
| **Study** | **LLM-type** | **model specification (detailed in original article)** | **dataset (detailed in original article)** | **Precision** | **Recall / Sensitivity** | **Specificity** | **F1** | **Accuracy** | **Krippendorff’s alpha** |
|  |  |  |  | Proportion of true positive results among all positive predictions | Proportion of true positive cases correctly identified among all actual positive cases. | Proportion of true negative cases correctly identified among all actual negative cases. | Harmonic mean of precision and recall, balancing their trade-off in a single metric. | Proportion of all correct predictions among the total number of cases. | Reliability measure assessing the agreement among raters (“humans consensus-based screening decisions” vs. “LLM classifications”), accounting for chance agreement. |
| Tran et al., 2024 | GPT 3.5 | Balanced Rule | Sommer et al, 2023 (23) | 0,50 | 0,81 | 0,57 | 0,62 | 0,66 | 0,34 |
| Tran et al., 2024 | GPT 3.5 | Balanced Rule | Sommer et al, 2023 (24) | 0,11 | 0,82 | 0,80 | 0,20 | 0,80 | 0,15 |
| Tran et al., 2024 | GPT 3.5 | Balanced Rule | S. Yaacoub, | 0,17 | 0,91 | 0,42 | 0,28 | 0,47 | 0,11 |
| Tran et al., 2024 | GPT 3.5 | Balanced Rule | Kiesswetter et al, 2023 (25) | 0,02 | 0,86 | 0,56 | 0,04 | 0,56 | 0,02 |
| Tran et al., 2024 | GPT 3.5 | Balanced Rule | Sbidian et al, 2023 (26) | 0,19 | 0,97 | 0,26 | 0,32 | 0,36 | 0,08 |
| Tran et al., 2024 | GPT 3.5 | Sensitive Rule | Sommer et al, 2023 (23) | 0,38 | 0,98 | 0,15 | 0,55 | 0,43 | 0,09 |
| Tran et al., 2024 | GPT 3.5 | Sensitive Rule | Sommer et al, 2023 (24) | 0,05 | 0,95 | 0,45 | 0,09 | 0,47 | 0,04 |
| Tran et al., 2024 | GPT 3.5 | Sensitive Rule | S. Yaacoub, | 0,12 | 1,00 | 0,02 | 0,21 | 0,13 | 0,00 |
| Tran et al., 2024 | GPT 3.5 | Sensitive Rule | Kiesswetter et al, 2023 (25) | 0,01 | 1,00 | 0,18 | 0,03 | 0,19 | 0,01 |
| Tran et al., 2024 | GPT 3.5 | Sensitive Rule | Sbidian et al, 2023 (26) | 0,16 | 1,00 | 0,05 | 0,27 | 0,19 | 0,02 |
| Robinson et al., 2023 | GPT 3.5 |  |  | 0,59 | 0,96 |  | 0,73 | 0,60 |  |
| Huotala et al., 2024 | GPT 3.5 |  |  | 0,65 | 0,18 |  | 0,28 |  |  |
| Kataoka et al., 2023 | GPT 3.5 |  | validation dataset 1 | 0,03 | 0,99 | 0,30 | 0,06 | 0,32 | -0,48 |
| Kataoka et al., 2023 | GPT 3.5 |  | validation dataset 2 | 0,19 | 0,92 | 0,44 | 0,31 | 0,50 | -0,08 |
| Syriani et al., 2023 | GPT 3.5 |  | RL4Se | 0,20 | 0,82 | 0,69 | 0,32 |  | 0,21 |
| Syriani et al., 2023 | GPT 3.5 |  | DSMLCompo | 0,13 | 0,87 | 0,67 | 0,23 |  | 0,15 |
| Syriani et al., 2023 | GPT 3.5 |  | MobileMDE | 0,51 | 0,33 | 0,93 | 0,40 |  | 0,30 |
| Syriani et al., 2023 | GPT 3.5 |  | MPM4CPS | 0,66 | 0,74 | 0,59 | 0,70 |  | 0,33 |
| Syriani et al., 2023 | GPT 3.5 |  | UpdateCollabMDE | 0,11 | 0,95 | 0,46 | 0,20 |  | 0,09 |
| Schopow et al., 2023 | GPT 3.5 |  | just titles, no abstracts | 0,65 | 1,00 | 0,50 | 0,79 | 0,74 | 0,49 |
| Schopow et al., 2023 | GPT 3.5 |  | just abstracts, no titles | 0,40 | 1,00 | 0,41 | 0,57 | 0,57 | 0,28 |
| Issaiy et al., 2024 | GPT 3.5 |  |  | 0,28 | 0,95 | 0,65 | 0,43 |  | 0,30 |
|  |  |  |  |  |  |  |  |  |  |
| Guo et al., 2024 | GPT 4.0 |  | IVM | 0,26 | 0,69 | 0,76 | 0,38 | 0,75 | 0,26 |
| Guo et al., 2024 | GPT 4.0 |  | SSRI | 0,12 | 0,97 | 0,95 | 0,22 | 0,95 | 0,21 |
| Guo et al., 2024 | GPT 4.0 |  | LPVR | 0,21 | 0,59 | 0,86 | 0,31 | 0,85 | 0,25 |
| Guo et al., 2024 | GPT 4.0 |  | RAYNAUDS | 0,14 | 0,83 | 0,97 | 0,23 | 0,97 | 0,22 |
| Guo et al., 2024 | GPT 4.0 |  | NOA | 0,16 | 0,78 | 0,90 | 0,26 | 0,90 | 0,23 |
| Guo et al., 2024 | GPT 4.0 |  | LLM | 0,12 | 1,00 | 0,94 | 0,22 | 0,94 | 0,21 |
| Wilkins, 2023 | GPT 4.0 |  |  |  | 0,71 | 0,89 |  | 0,84 |  |
| Huotala et al., 2024 | GPT 4.0 |  |  | 0,50 | 0,42 |  | 0,45 |  |  |
| Cai et al., 2023 | GPT 4.0 | Strategy 3 | Glioma | 0,13 | 1,00 |  | 0,22 |  |  |
| Cai et al., 2023 | GPT 4.0 | Strategy 3 | Inflammatory | 0,15 | 1,00 |  | 0,27 |  |  |
| Cai et al., 2023 | GPT 4.0 | Strategy 3 | Diabetes | 0,13 | 0,78 |  | 0,22 |  |  |
| Cai et al., 2023 | GPT 4.0 | Strategy 3 | Sarcopenia | 0,24 | 0,90 |  | 0,38 |  |  |
| Kataoka et al., 2023 | GPT 4.0 |  | validation dataset 1 | 0,06 | 0,98 | 0,68 | 0,12 | 0,68 | -0,07 |
| Kataoka et al., 2023 | GPT 4.0 |  | validation dataset 2 | 0,30 | 0,81 | 0,74 | 0,44 | 0,75 | 0,28 |
| Urrutia et al.,2023 | GPT 4.0 | 4-shots CoT | SAC | 0,65 | 0,63 |  | 0,63 |  |  |
| Yang et al., 2024 | GPT 4.0 |  | SARS-CoV-2 incl reviews | 0,95 | 0,71 | 0,98 | 0,81 | 0,89 | 0,74 |
| Yang et al., 2024 | GPT 4.0 |  | Nipah including reviews | 0,73 | 0,75 | 0,91 | 0,74 | 0,87 | 0,66 |
| Khraisha et al., 2024 | GPT 4.0 |  |  |  | 0,42 | 0,92 |  | 0,67 |  |
|  |  |  |  |  |  |  |  |  |  |
| Wang et al., 2024 | Alpaca |  |  |  | 0,91 |  |  |  |  |
| Robinson et al., 2023 | Llama |  |  | 0,83 | 0,72 |  | 0,77 | 0,74 |  |
| Wang et al., 2024 | Llama |  |  |  | 0,89 |  |  |  |  |
| Robinson et al., 2023 | Guanaco |  |  | 0,73 | 0,84 |  | 0,78 | 0,67 |  |
